# A novel predictive model for the recurrence of pediatric alopecia areata by bioinformatics analysis and a single-center prospective study

**DOI:** 10.3389/fmed.2023.1189134

**Published:** 2023-06-08

**Authors:** Yuanquan Zheng, Yingli Nie, Jingjing Lu, Hong Yi, Guili Fu

**Affiliations:** Department of Dermatology, Wuhan Children’s Hospital (Wuhan Maternal and Child Healthcare Hospital), Tongji Medical College, Huazhong University of Science and Technology, Wuhan, China

**Keywords:** alopecia areata, biomarker, immune, prognosis, logistic regression

## Abstract

**Background:**

Alopecia areata (AA) is a disease featured by recurrent, non-scarring hair loss with a variety of clinical manifestations. The outcome of AA patients varies greatly. When they progress to the subtypes of alopecia totalis (AT) or alopecia universalis (AU), the outcome is unfavorable. Therefore, identifying clinically available biomarkers that predict the risk of AA recurrence could improve the prognosis for AA patients.

**Methods:**

In this study, we conducted weighted gene co-expression network analysis (WGCNA) and functional annotation analysis to identify key genes that correlated to the severity of AA. Then, 80 AA children were enrolled at the Department of Dermatology, Wuhan Children’s Hospital between January 2020 to December 2020. Clinical information and serum samples were collected before and after treatment. And the serum level of proteins coded by key genes were quantitatively detected by ELISA. Moreover, 40 serum samples of healthy children from the Department of Health Care, Wuhan Children’s Hospital were used for healthy control.

**Results:**

We identified four key genes that significantly increased (*CD8A, PRF1*, and *XCL1*) or decreased (*BMP2*) in AA tissues, especially in the subtypes of AT and AU. Then, the serum levels of these markers in different groups of AA patients were detected to validate the results of bioinformatics analysis. Similarly, the serum levels of these markers were found remarkedly correlated with the Severity of Alopecia Tool (SALT) score. Finally, a prediction model that combined multiple markers was established by conducting a logistic regression analysis.

**Conclusion:**

In the present study, we construct a novel model based on serum levels of *BMP2, CD8A, PRF1*, and *XCL1*, which served as a potential non-invasive prognostic biomarker for forecasting the recurrence of AA patients with high accuracy.

## 1. Introduction

Alopecia areata (AA) is characterized by chronic, recurrent and non-scarring hair loss, with a 2% lifetime risk (1). According to the intensity and area of hair loss, AA is divided into three subtypes: alopecia areata in patches (AAP), alopecia totalis (AT), and alopecia universalis (AU) (2). The prognosis in each AA patient is variable and unpredictable, and the extent of hair loss is the most significant prognostic factor. Although numbers treatments for AA have been introduced, the long-term efficacy and the therapeutic response varies widely (3). Therefore, it is critical to establish a novel predictive tool to anticipate the recurrence of AA that might lead to more individualized interventions for AA patients and prevent the recurrence of AA.

Our previous study found that the over-activating of immune response and the dysfunction of epidermis and hair development processes are two essential factors for AA occurrence and development. We constructed high-accuracy biomarkers by using machine learning (ML) algorithms based on four key genes (*CD28*, *HOXC13*, *KRTAP1-3*, and *GPRC5D*) involved in immune response and epidermis and hair development processes (4). However, the proteins coded by these genes cannot be detected by non-invasive methods, which limited the clinical application of this model.

In the present study, we conducted bioinformatics analysis and identify four key genes (*CD8A, PRF1, XCL1*, and *BMP2*) that encode plasma proteins associated with the severity of AA. Then, 80 AA children were enrolled at the Department of Dermatology, Wuhan Children’s Hospital between January 2020 to December 2020. Clinical information and serum samples were collected before and after treatment. And the serum level of proteins coded by key genes was quantitatively detected by ELISA. Moreover, 40 serum samples of healthy children from the Department Health Care, Wuhan Children’s Hospital were used for healthy control. The protein levels of these markers were found remarkedly correlation with Severity of Alopecia Tool (SALT) score. Moreover, a prediction model that combined these markers was established by conducting a logistic regression analysis.

## 2. Materials and methods

### 2.1. Bioinformatical analysis

#### 2.1.1. Data source and preprocessing

Datasets related to AA were downloaded from NCBI-GEO,^1^ with an accession number of GSE68801 (5), including 60 AA skin samples and 62 normal skin samples.

#### 2.1.2. Weighted gene co-expression network construction and interesting module detection

Weighted gene co-expression network construction and module identification of DEGs were performed following the protocols of weighted gene co-expression network analysis (WGCNA) (6), described previously (7). Briefly, every pairwise gene–gene relationship was calculated by a gene coexpression similarity in the first step. Then, a “soft” power adjacency function was utilized to construct the adjacency matrix and topological overlap matrix (TOM). “Gene modules,” groups of genes that have high topological overlap, were identified using hierarchical clustering with a dissimilarity measure (1-TOM).

The correlations between modules and clinical features were identified by Pearson correlation tests to identify clinically meaningful modules. The modules that exhibited a high correlation with prognostic features were selected as interesting modules to be further studied.

#### 2.1.3. Functional annotation analysis

Metascape database^2^ (8) was used to annotate the function of interesting modules identified by WGCNA. *p* < 0.05 was set as the cut-off criterion.

### 2.2. Single-center prospective study

#### 2.2.1. Study population and design

A single-center prospective study was performed in the Department of Dermatology, Wuhan Children’s Hospital from January 2020 to December 2020. The study was performed following local ethics committee approval (Wuhan Children’s Hospital, Tongji Medical College, Huazhong University of Science and Technology), and written informed consent was obtained from all participants. Inclusion criteria were patients with first-visit and mild-to-severe AA. Exclusion criteria were: (1) patients who suffer from other hair diseases such as telogen effluvium, trichotillomania, scarring alopecia or tinea capitis, and (2) being refractory to previous therapy. The clinical characteristics (including gender, age, and classification (based on SALT score, S1: <25%, S2: 25–49%, S3–S5: 50–100%)) and serum sample for detecting *CD8A, PRF1, XCL1*, and *BMP2* were collected at the basal visit and at the 6-month follow-up visit. The treatment of AA is tailored according to the age of the patient and the extent of the disease. The combination of midpotent or potent topical corticosteroid (such as mometasone furoate cream or halometasone cream) once a day with 5% topical minoxidil solution twice a day was the standard therapy for the group of S1. In addition to topical corticosteroids and minoxidil, 308 nm excimer laser twice a week were offered for the group of S2. Other therapeutic options were offered for the group of S3-5, including potent topical corticosteroid under occlusion at night, 5% topical minoxidil twice a day, and oral compound glycyrrhizin tablet. Systemic corticosteroid used in AA is less-favored option because of the side effect profile, include hyperglycemia, osteoporosis, cataracts, immunosuppression, mood changes, obesity, dysmenorrhea, acne, and Cushing syndrome. Compound glycyrrhizin tablets have anti-inflammatory effects similar to corticosteroid without corticosteroid-related side effects (9). Therefore, we choose oral compound glycyrrhizin tablet for the systemic therapy for the group of S3-5.

Three categories of outcome were defined: ineffective as <30% of regrowth, improve as 30–90% of regrowth, and cure as >90% of regrowth. Moreover, serum samples of healthy children from the Department of Health Care, Wuhan Children’s Hospital were used for healthy control.

#### 2.2.2. Enzyme-linked immunosorbent assay (ELISA)

ELISA kits were used to detect *CD8A* (Bioswamp, cat. No: HM12842), *PRF1* (Bioswamp, cat. No: HM10300), *XCL1* (Abcam, ab264620), and *BMP2* (Bioswamp, cat. No: HM10833) according to the manufacturer’s protocols.

#### 2.2.3. Statistical analysis

All the computational and statistical analyses were performed using R programming^3^ and SPSS 22.0 (IBM Corp., Armonk, NY, United States). Student’s *t*-test was used to compare two groups with normally distributed variables. For comparisons of three groups, one-way ANOVA analysis and Kruskal-Wallis tests of variance were used as parametric and nonparametric methods, respectively. Chi-square was used to evaluate the differences between groups for categorical variables. Pearson’s correlation analysis was used to compute the correlation coefficients. A prediction model that combined four markers for the recurrence of AA was further established by performing a logistic regression analysis. The area under the receiver operating characteristic (ROC) curve was utilized to evaluate the predictive value of the markers and the prediction model. *p*-value of <0.05 was selected as the threshold of statistical significance.

## 3. Results

### 3.1. Bioinformatical analysis

#### 3.1.1. Coexpression network construction and interesting module detection

**Figure 1:**
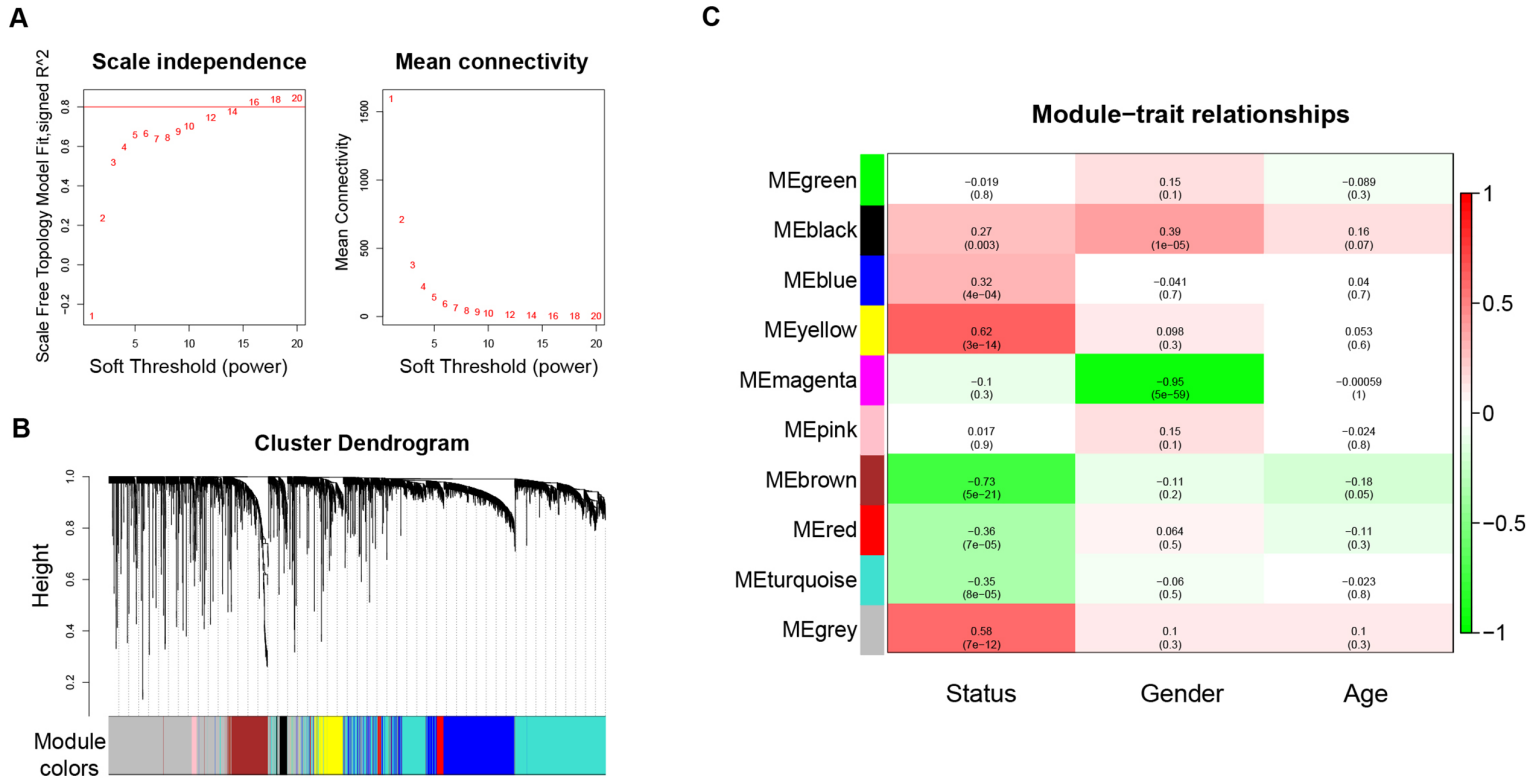
WGCNA network and module detection. **(A)** Power 14 was chosen as the soft-thresholding power. **(B)** Cluster dendrogram and module assignment for modules from WGCNA. **(C)** Correlation matrix for eigengene values and clinical features. Each cell includes the corresponding correlations and the *p*-values. WGCNA: weighted gene co-expression network analysis.

WGCNA was performed based on the top 10% genes with the most expression variance (5300) among the 122 skin samples. The connectivity between the genes in the gene network formed a scale-free network distribution when the soft-threshold power b was set to 14 (Figure 1A). Then, 9 coexpression modules were identified and represented by different colors (Figure 1B). The correlations between modules and clinical features, such as Status, gender, and age were calculated. The yellow module was highly positively correlated with Status (r = 0.62, *p* = 3 × 10^−14^), and the brown module was highly negatively correlated with Status (r = −0.73, *p* = 5 × 10^−21^) (Figure 1C). Thus, the yellow and brown modules were selected as interesting modules to be studied in subsequent analyses.

#### 3.1.2. Annotation enrichment analysis

**Figure 2:**
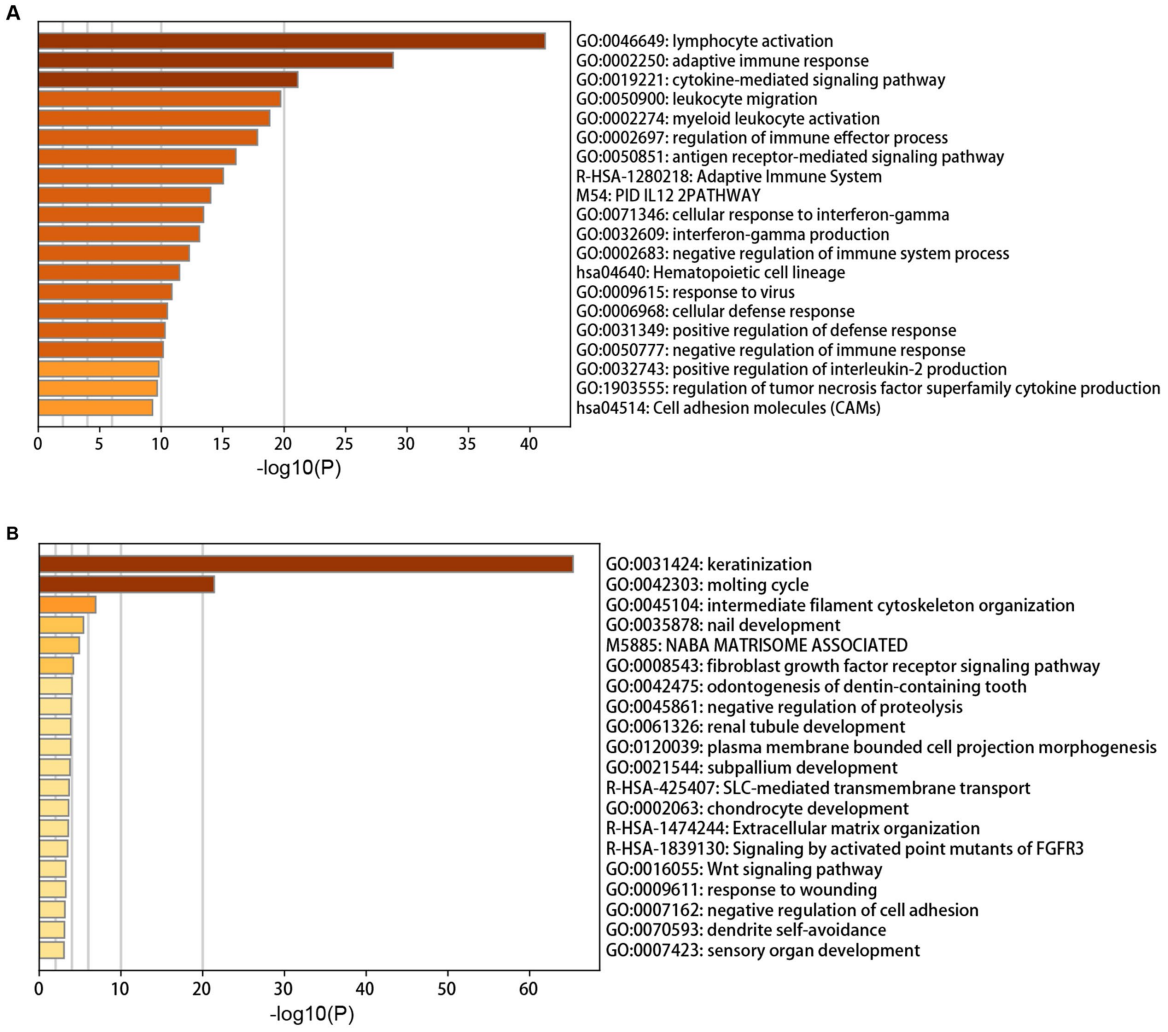
Annotation enrichment analysis of interesting modules. **(A)** Functional enrichment analysis of genes in the yellow module. **(B)** Functional enrichment analysis of genes in the brown module.

The annotation enrichment analysis showed that the genes in the yellow module were significantly enriched in immune response pathways, especially in lymphocyte activation, adaptive immune response, and cytokine-mediated signaling pathway (Figure 2A). However, the biological processes of the brown module were significantly enriched in skin and hair related processes, especially in keratinization and molting cycle (Figure 2B).

#### 3.1.3. Identification of key genes for predicting the risk of AA progression

**Figure 3:**
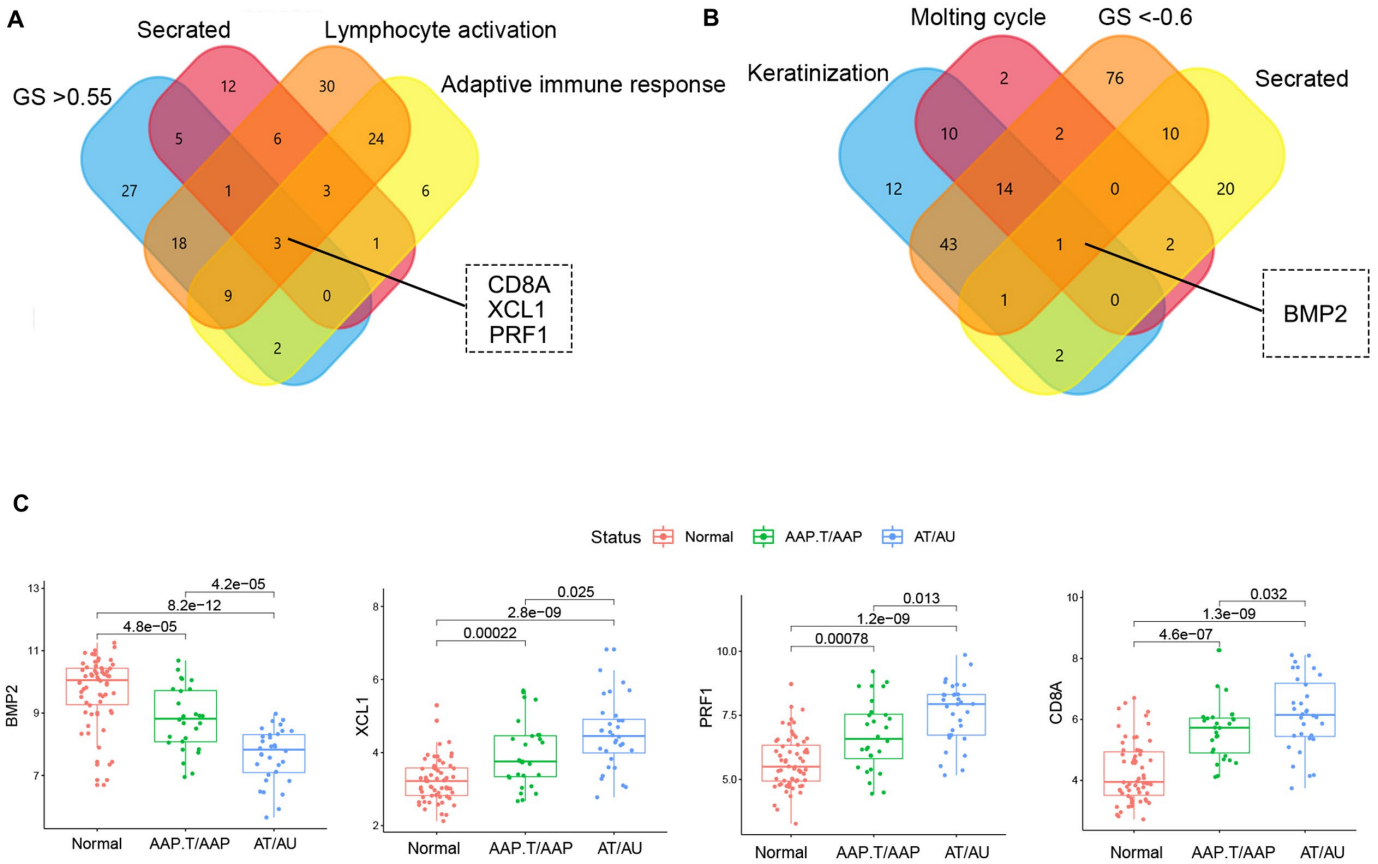
The identification of key genes. The Venn plots of the candidate marker identification in yellow **(A)** and brown **(B)** modules. **(C)** The expression levels of key genes in different AA groups.

The genes with high gene significance (GS > 0.6 in the yellow module or GS < −0.6 in the yellow module), encoding secreted proteins, and belonging to the most enriched biological processes (lymphocyte activation, adaptive immune response, keratinization, and molting cycle) were selected as candidate key genes (Figures 3A,B). Finally, *BMP2, CD8A, PRF1*, and *XCL1* were chosen as key genes. Among them, *CD8A, PRF1*, and *XCL1* were markedly increased while *BMP2* was significantly decreased in AA tissues, especially in the subtypes of AT and AU (Figure 3C).

### 3.2. Single-center prospective study

#### 3.2.1. Demographic and clinical features

The demographic features of patients are summarized in Table 1. A total of 80 AA patients met the criteria and were included in the study [male, 46.25% (*n* = 37); female, 53.75% (*n* = 43); mean ± SD age = 5.92 ± 3.15 years]. AA patients of S1 accounts for 42.50% (*n* = 34), S2 accounts for 15.00% (*n* = 12), and S3-5 accounts for 42.50% (*n* = 34). At the 6-month follow-up visit, A complete regrowth was observed in 22 patients (27.5%) whereas a partial regrowth in 52 patients (65.00%). In 6 patients (7.50%), no treatment response was observed.

**Table 1:** Basic features of the patient population (*n* = 80).

Characteristics	S1 (*n* = 34)	S2 (*n* = 12)	S3-5 (*n* = 34)	*p* value
Gender				0.226
Male	19 (55.88%)	6 (50.00%)	12 (35.29%)	
Female	15 (44.12%)	6 (50.00%)	22 (64.71%)	
Age, mean ± SD	6.76 ± 3.23	5.83 ± 3.00	5.12 ± 2.84	0.06
Outcomes				0.007
Ineffective	1 (2.94%)	2 (16.67%)	3 (8.82%)	
Improve	9 (26.47)	7 (58.33%)	25 (73.53%)	
Cure	24 (70.59%)	3 (25.00%)	5 (17.65%)	
Recurrence				0.002
Yes	10 (29.42%)	0 (0.00%)	1 (2.94%)	
No	24 (70.58%)	12 (100.00%)	33 (97.06%)	

We further compared the characteristics of AA patients in different severity groups. As shown in Table 2, there was no difference in gender and age among the three groups. However, significant differences were found in outcome and recurrence. Patients in the S1 group presented the best outcome with a 70.59% cure rate, and the highest recurrence rate of 29.42%. Patients in the S2 group with the highest ineffective of 16.67% and lowest recurrence rate of 0%.

**Table 2:** Characteristics of the patient population in different groups.

Characteristics	Patients
Gender	
Male	37 (46.25%)
Female	43 (53.75%)
Age, mean ± SD	5.92 ± 3.15
SALT score, median (P25, P75)	33 (13, 64)
Classification	
S1	34 (42.50%)
S2	12 (15.00%)
S3-S5	34 (42.50%)
Outcomes	
Ineffective	6 (7.50%)
Improve	52 (65.00%)
Cure	22 (27.50%)
Recurrence	
Yes	11 (13.85%)
No	69 (86.25%)

#### 3.2.2. Correlation of serum levels of *BMP2, CD8A, PRF1*, and *XCL1* in AA patients

**Figure 4:**
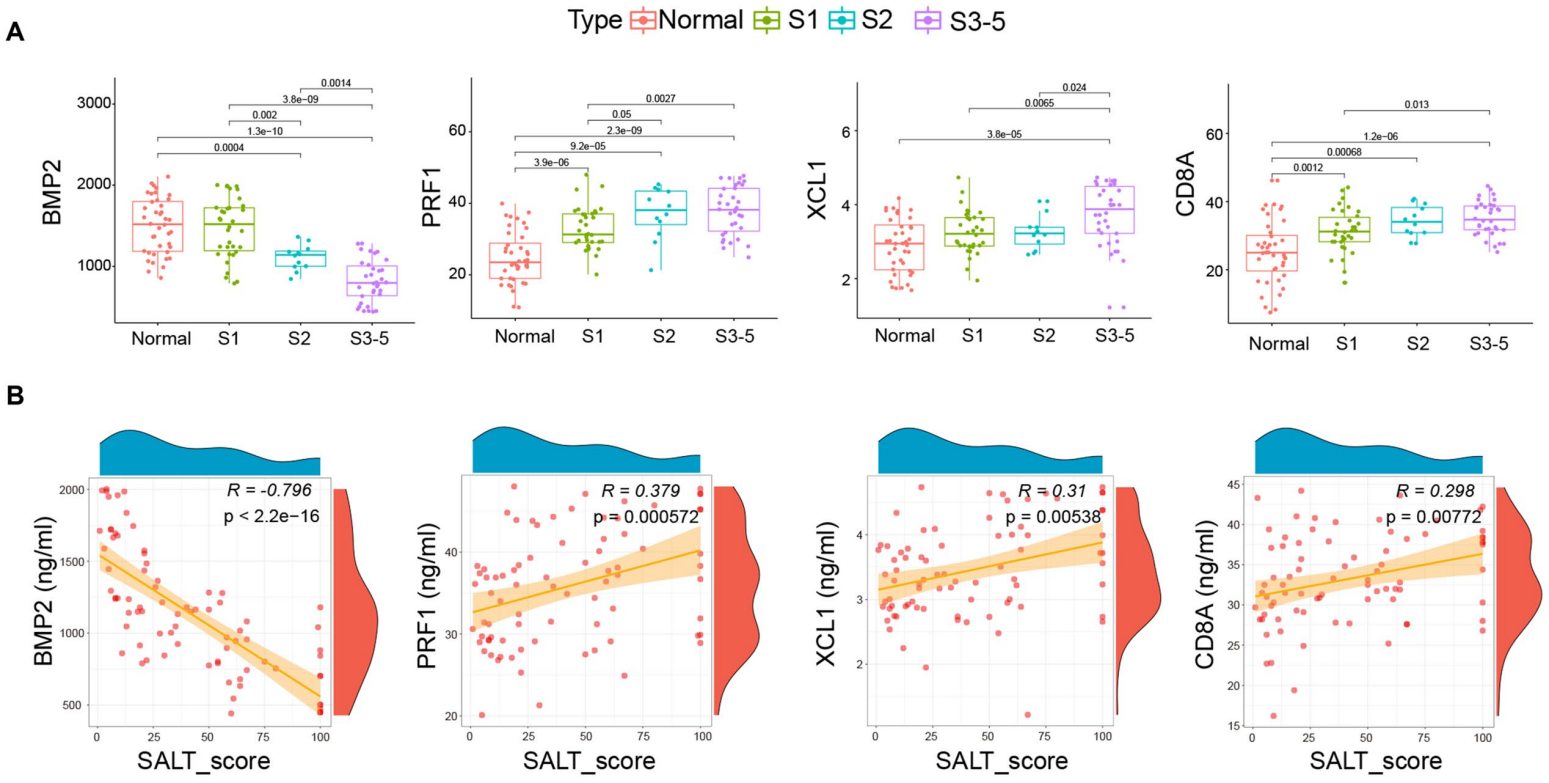
Correlation of *BMP2, CD8A, PRF1*, and *XCL1* with pediatric AA. **(A)** Serum level of the four markers in health individuals and AA patient groups. **(B)** The correlation of the four markers with the SALT scores. SALT: Severity of Alopecia Tool. Normal: Health individuals.

As shown in Figure 4A, similar to the expression profile of *BMP2, CD8A, PRF1*, and *XCL1* in AA patients from GSE68801, the serum levels of BMP2 were markedly decreased in AA patients, especially in S3-5 group. However, the serum levels of *CD8A, PRF1*, and *XCL1* were the contrary. The correlation analysis further demonstrated that the serum level of *BMP2* was highly negatively correlated with SALT score (*R* = -0.796, *p* < 2.2e-16). *CD8A* (*R* = 0.298, *p* = 0.00772), *PRF1* (*R* = 0.379, *p* = 0.000572), and *XCL1* (*R* = 0.31, *p* = 0.00538) were significantly negatively correlated with the SALT score (Figure 4B).

**Figure 5:**
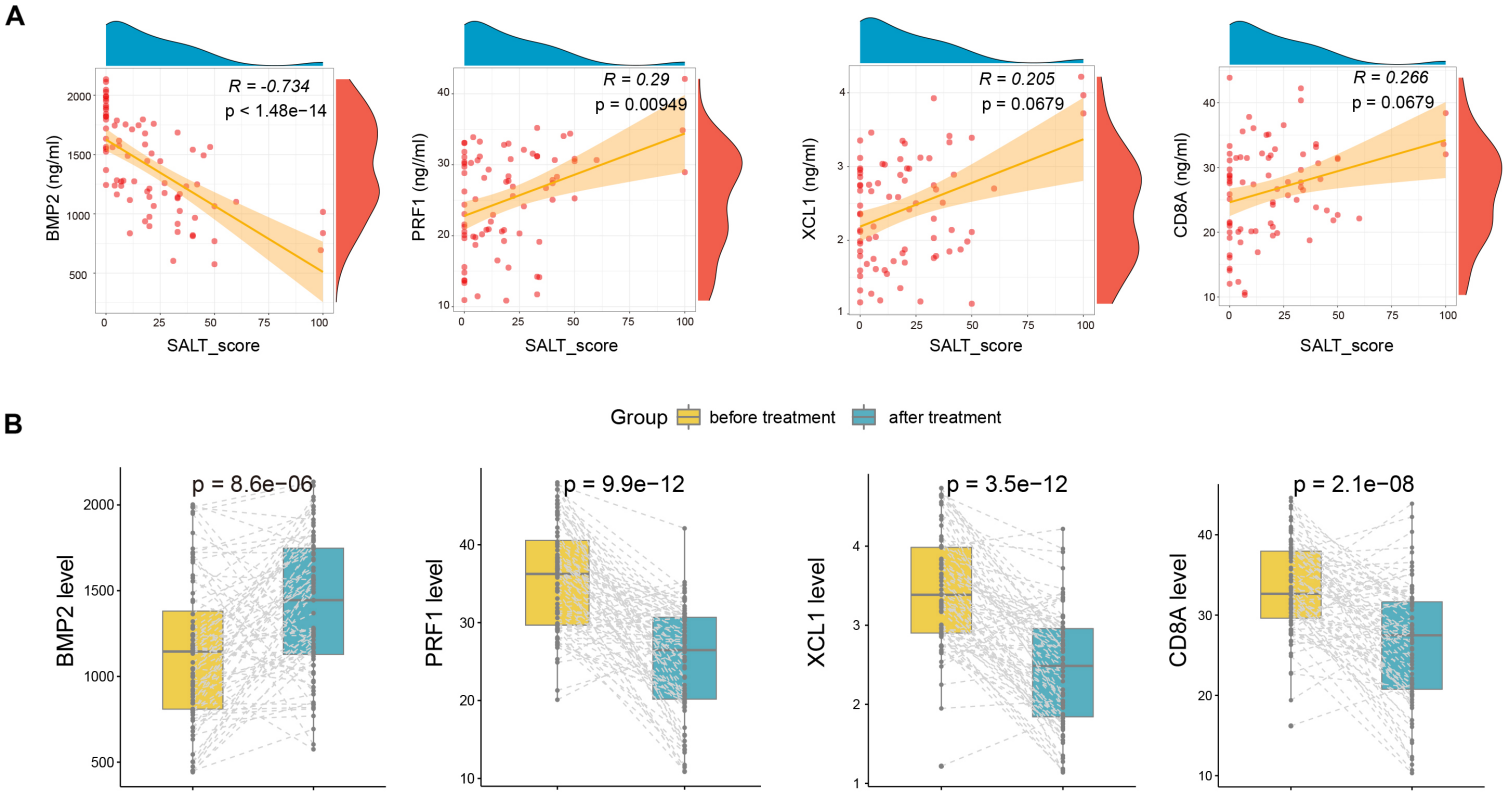
The alteration of serum *BMP2, CD8A, PRF1*, and *XCL1* after treatment. **(A)** The correlation of the four markers with SALT scores after treatment. **(B)** The alteration of serum *BMP2, CD8A, PRF1*, and *XCL1* after treatment.

Moreover, we also detected the serum levels of the four markers after treatment. As shown in Figure 5A, the serum levels of BMP2(*R* = −0.734, *p* < 1.48e-14) and PRF1 (*R* = 0.29, *p* = 0.000949) were also significantly correlated with and SALT score after treatment. Furthermore, after treatment, serum levels of BMP2 increased markedly, while PRF1, XCL1, and CD8A declined dramatically (Figure 5B).

#### 3.2.3. Predictive model for the recurrence of AA

To construct a predictive model for forecasting the recurrence of AA. *BMP2, PRF1, XCL1*, and *CD8A* were included in the logistic regression model. There was no multicollinearity was found in the markers with variance inflation factor (VIF) <10. The prediction model was as follows:


$$\begin{array}{c} \text{Logit}\;\left(P\right)=-8.827+0.004\;\left(BMP2\right)-0.094\;\left(PRF1\right)\\ +0.613\left(XCL1\right)+0.091\left(CD8A\right). \end{array}$$

**Figure 6:**
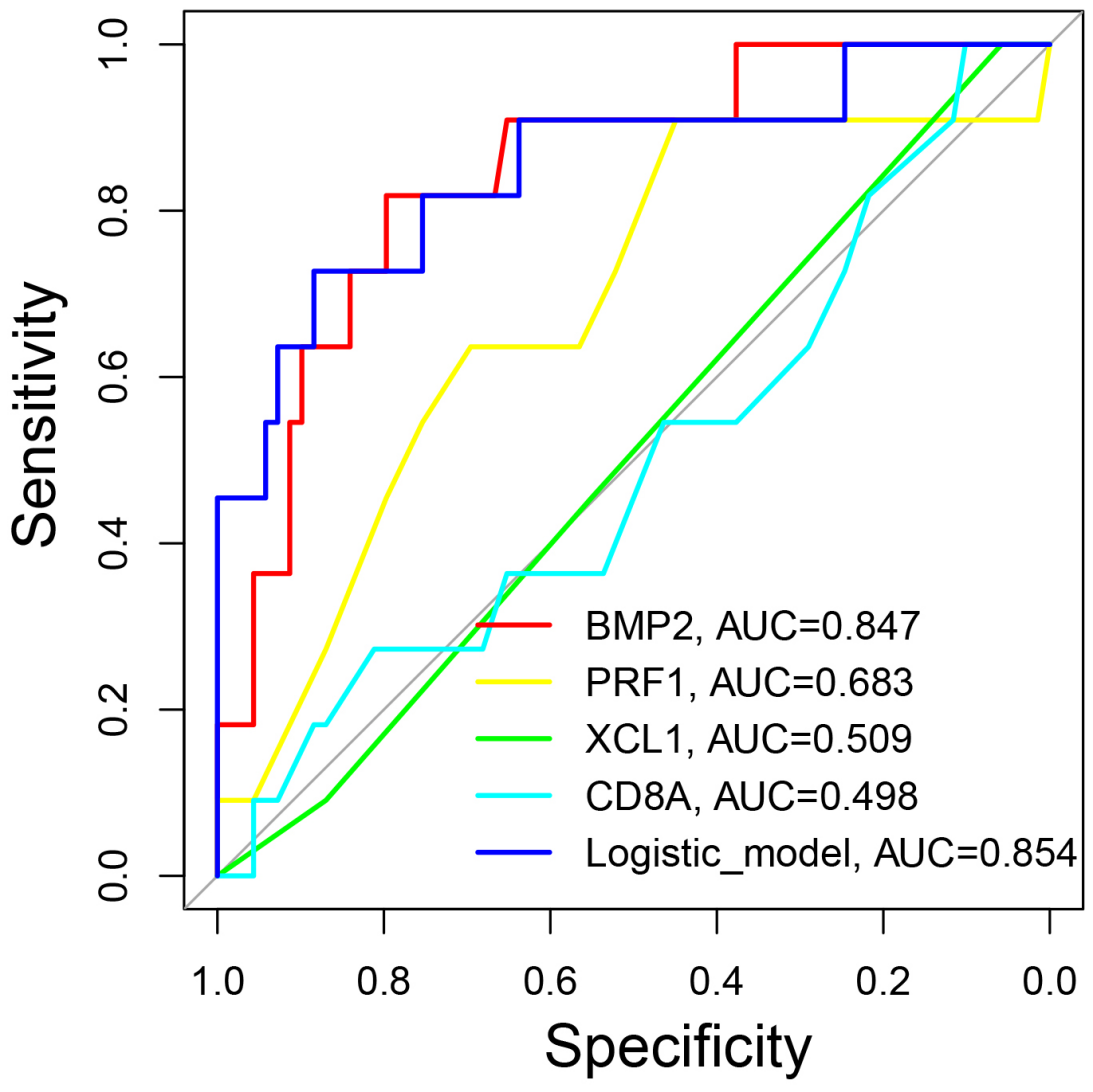
The predictive significance of the model for forecasting the recurrence of pediatric AA. ROC curves of the model and individual marker for predicting the recurrence of pediatric AA. ROC: the receiver operating characteristic. AUC: area under the curve.

The ROC curves for the markers and the logistic regression model for predicting AA recurrence was depicted in Figure 6. The prediction model had the highest AUC value of 0.854 (*p* < 0.001), when compared to the values of 0.847 for BMP2 (*p* < 0.001), 0.683 for PRF1 (*p* = 0.045), 0.509 for XCL1 (*p* = 0.716), and 0.498 for CD8A (*p* = 0.989). Moreover, the optimum cut-off value of the model is 0.2148 with a max Youden index of the model is 0.611, a sensitivity of 0.727, and a specificity of 0.884.

## 4. Discussion

AA is an autoimmune disease characterized by relapsing–remitting remissions that may persist if hair loss is severe. Hair follicle damage attributed to the breakdown of the immune privilege in hair follicle has been thought to be a major trigger of AA, which lead to non-scarring hair loss without obvious epidermal changes (10). Recently, increasing studies aimed to stratify AA patient by constructing prognostic models based on gene expression pattern and clinical characteristics due to the variable and unpredictable prognosis of AA patients. Lee et al. identified the topographic phenotypes of AA using cluster analysis and established a prediction model and grading system for predicting AA prognosis, which may help clinicians to establish better treatments (11). Our previous study established high accuracy models for predicting the risk of AA patients progressing to AT or AU based on four key AA associated genes (*CD28, HOXC13, KRTAP1-3*, and *GPRC5D*) through ML algorithms (4).

Similar to our previous study (4), in the present study, we found that genes enriched in immune-related biological processes and pathways were highly positively correlated with the severity of AA. However, genes involved in epidermis and hair development processes were decreased significantly in patients with severe subtypes of AA. Indicated that the over-activating of immune response and the dysfunction of the epidermis and hair development processes play important roles in the progress of AA. Among the four key genes, *CD8A, PRF1*, and *XCL1* are involved in immune processes, while BMP2 played important roles in the hair cycle and keratinization process.

The alterations of their expression levels play central roles in the occurrence and progression of AA. Bone morphogenetic protein 2 (*BMP2*) is a member of the transforming growth factor-*β* (*TGF-β*) family, which plays key roles in the activation of hair follicle niches by neural stem cell extracts to promote hair growth (12), and promotes hair follicle stem cells differentiation by inducing autophagy (13). *CD8A* is the unique surface glycoprotein of CD8^+^ T cells. The upregulation of the expression level of *CD8A* in AA samples indicated the increased infiltration of CD8^+^ T cells in AA samples. It is now generally accepted that the collapse of the hair follicles immune privilege is the crucial precondition for the development of AA. Moreover, CD8^+^ T cells are the key effector cells in human AA. A previous study found that cytotoxic CD8^+^NKG2D^+^ T cells, a type of skin-infiltrating CD8^+^ T cells in mice, are both necessary and sufficient for the induction of AA in mouse models of disease (14). The excessive activity of CD8^+^NKG2D^+^ T cells will lead to an IFN-γ “storm,” which induces a damage response pattern of the AA in normal scalp skin both in humans and mice (15). *XCL1* is a member of C-class chemokines, which are expressed by various immune cells, including activated CD8+ T cells. *XCL1* elicits its chemotactic function by binding to the receptor called *XCR1*. *XCR1* is expressed by a dendritic cell (DC) subpopulation. The XCL1-XCR1-mediated chemoattraction may facilitate antigen presentation from DCs to CD8+ T cells and promote the proliferation and differentiation of CD8^+^ T cells, leading to the release of IFN-γ (16). Perforin (*PRF1*) is primarily expressed in NK cells and cytotoxic T lymphocytes (CTLs) (17), which acts as the main effector for these cells to exert cytotoxic capacity. PRF1 is a pore-forming protein that polymerizes and forms transmembrane channels in plasma membrane lipid bilayers. Moreover, *PRF1* is the only currently known delivery that delivers granzymes into the cytosol of target cells, which initiate and directly mediate apoptosis of target cells (18). However, the role of *PRF1* in the progression of AA remains unclear.

To further verify the prognostic effect of these markers, we conducted a prospective study and detected the serum level of *BMP2, CD8A, PRF1*, and *XCL1* in pediatric AA patients. Similar to the bioinformatics analysis, we found that the serum levels of *BMP2* were significantly decreased in AA patients, especially in the severe subgroup. The obvious elevations were detected of *CD8A, PRF1*, and *XCL1* in AA patients, especially in the severe subgroup. Since the close association of these four markers with the progression and severity of AA, these markers can act as promising biomarkers to build a predictive mode for forecasting the prognosis of AA. In the present study, we construct a model utilizing logistic regression analysis based on *BMP2, CD8A, PRF1*, and *XCL1*. The model performed well in predicting the recurrence of pediatric AA with the AUC value of 0.854, sensitivity value of 0.727, and specificity value of 0.884.

Inevitably, the present study has some innate limitations. Firstly, the microarray data used for bioinformatic analysis was obtained from adult AA skin samples, while the gene expression pattern of adult AA may be different from that of pediatric AA. Secondly, although we quantitatively analyze the serum levels of the four markers, the normal range for the serum levels of the proteins remains unclear due to the insufficiency of samples. At last, the cohort of the current study consisted of only 80 samples. Therefore, large-scale, multicenter studies are needed to verify our results.

## 5. Conclusion

*BMP2, CD8A, PRF1*, and *XCL1* presented highly association with the severity of pediatric AA. Implementation of logistic regression analysis based on the four markers to establish a predictive model may help to early identify high-risk recurrent pediatric AA patients and lead to more individualized interventions for them to prevent the recurrence of AA.

## Data availability statement

The datasets presented in this study can be found in online repositories. The names of the repository/repositories and accession number(s) can be found in the article/supplementary material.

## Ethics statement

The studies involving human participants were reviewed and approved by the Ethics Committee of Tongji Medical College, Huazhong University of science and technology (Wuhan, China). Written informed consent to participate in this study was provided by the participants’ legal guardian/next of kin.

## Author contributions

YN and YZ participated with substantial contributions in the conception and design of the work. JL and HY acquired, analyzed, and interpreted data. YZ and GF drafted and critically revised the work, obtained funding, and supervision. All authors contributed to the article and approved the submitted version.

## Funding

This study was supported by the Scientific research project of Wuhan Municipal Health Commission (No. WX20C09).

## Conflict of interest

The authors declare that the research was conducted in the absence of any commercial or financial relationships that could be construed as a potential conflict of interest.

## Publisher’s note

All claims expressed in this article are solely those of the authors and do not necessarily represent those of their affiliated organizations, or those of the publisher, the editors and the reviewers. Any product that may be evaluated in this article, or claim that may be made by its manufacturer, is not guaranteed or endorsed by the publisher.

## Footnotes

^1^ http://www.ncbi.nlm.nih.gov/geo

^2^ http://metascape.org

^3^ https://www.r-project.org/
